# Cytosolic Delivery of a Bithiophene Derivative via Polymersomes Kills *Trypanosoma cruzi* Amastigotes and Modulates the Inflammatory Response

**DOI:** 10.1021/acsanm.5c05104

**Published:** 2026-02-14

**Authors:** Rayanne Regina Beltrame Machado, Débora B Scariot, Amanda Beatriz Kawano Bakoshi, El Hadji Arona Mbaye, Sultan Almunif, Swagat Sharma, Deysiane Lima Salvador, Caroline Fortuna, Sueli de Oliveira Silva Lautenschlager, Tânia Ueda Nakamura, Maria Helena Sarragiotto, Danielle Lazarin Bidóia, Evan Scott, Celso Vataru Nakamura

**Affiliations:** † Laboratório de Inovação Tecnológica no Desenvolvimento de Fármacos e Cosméticos, Departamento de Ciências Básica da Saúde, Universidade Estadual de Maringá (UEM), Maringá, Paraná 87020-900, Brasil; ‡ Programa de Pós-graduação em Química, Departamento de Química, Universidade Estadual de Maringá (UEM), Maringá, Paraná 87020-900, Brasil; § Department of Biomedical Engineering, Chemistry of Life Processes Institute, 3270 Northwestern University, Evanston, Illinois 60208, United States; ∥ Department of Biomedical Engineering, NanoSTAR Institute, University of Virginia School of Medicine, Charlottesville, Virginia 22903, United States

**Keywords:** Chagas disease, polymersomes, bithiophene derivative, amastigotes, drug delivery, immunomodulation

## Abstract

Chagas disease, caused by the protozoan *Trypanosoma cruzi*, is an infectious illness that progresses through acute, indeterminate, and chronic phases. The acute phase often presents mild symptoms, followed by an asymptomatic indeterminate phase. In some cases, the disease advances to chronic Chagas cardiomyopathy, driven by sustained inflammation. Current treatments, benznidazole and nifurtimox, have limited efficacy in this stage and are associated with significant toxicity, highlighting the need for better therapies. PEG-*b*-PPS loaded with **BTAc**, exhibited favorable physicochemical properties, including high stability and an average size of 120 nm, suitable for efficient uptake by phagocytic cells such as macrophages. **BTAc**-loaded polymersomes showed enhanced efficacy against intracellular amastigotes of three *T. cruzi* strains (CL Brener, Brazil and Y) with IC_50_ values of 6.17, 24.01, and 30.68 μg/mL, respectively. The formulation simultaneously reduced proinflammatory cytokines to basal levels, suggesting an immunomodulatory potential. Importantly, Förster Resonance Energy Transfer analysis confirmed that **BTAc**-loaded polymersomes remain intact upon cellular uptake, escape the endosomal compartment, and release their payload directly into the cytosol, which is the intracellular niche where amastigotes persist during chronic infection. These findings underscore the potential of this nanocarrier system as an innovative approach that combines targeted antitrypanosomal therapy with enhanced immunomodulation, offering a promising strategy for the treatment of chronic Chagas disease.

## Graphical abstract

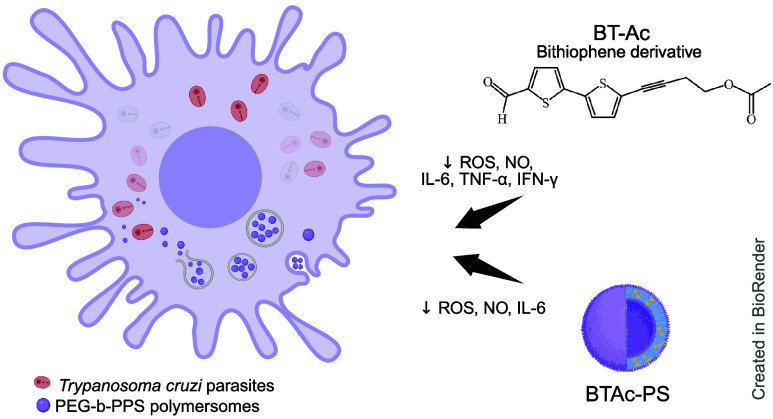

## Introduction

In recent years, nanoparticles have emerged as a highly effective strategy for enhancing drug delivery, offering targeted and controlled release mechanisms.^1^ These nanoscale carriers can be composed of metallic, polymeric, or lipid-based materials, each presenting unique advantages. Vesicular nanoparticles, such as liposomes and polymeric synthetic nanocarriers (SNC), can encapsulate hydrophobic and hydrophilic drugs. Among these, SNC demonstrates notable advantages over liposomes, including greater structural stability and controlled release *in vivo*.^2,3^ Additionally, SNC offer superior potential for targeted delivery and chemical functionalization, making them a promising platform for advanced therapeutic applications.^4^

Poly­(ethylene glycol)-*block*-poly­(propylene sulfide) (PEG-*b*-PPS) is an oxidation-sensitive amphiphilic copolymer composed of a hydrophilic polyethylene glycol (PEG) block and a hydrophobic polypropylene sulfide (PPS) block, which enables the formation of bilayer structures.^5^ This copolymeric platform has been applied in various disease models due to its capacity to target immune cells such as macrophages and dendritic cells.^6–9^ Depending on the hydrophilic-to-hydrophobic ratio of the PEG-*b*-PPS copolymer, different nanocarrier morphologies can be achieved, including micelles, polymersomes, and filomicelles.^10–12^

Nanocarrier-based approaches have previously been explored for the treatment of Chagas disease (CD).^13–15^ In addition, our group has recently reported the antitrypanosomal activity of a new synthetic compound, 4-(5′-formyl-[2,2′-bithiophen]-5-yl)­but-3-yn-1-yl acetate (**BTAc**). This compound belongs to the bithiophene class, which is well-known for its anti-inflammatory properties.^16^ **BTAc** exhibits moderate water solubility and induces redox imbalance and cell death in the main evolutionary forms of *Trypanosoma cruzi in vitro*.^17^ Overall, bithiophenes are recognized as effective antitrypanosomatid agents.^18^

CD, caused by the obligate intracellular parasite *T. cruzi*, is a neglected tropical disease endemic to Latin America, but it has become increasingly prevalent worldwide due to migration and globalization.^19^ Currently, it is estimated that 10,000 deaths occur every year, and around 7 million people are infected globally.^20^ Triatomine bugs primarily transmit parasites, but transmission can also occur via contaminated food, blood transfusion, organ transplantation, or congenitally. The infection begins when the parasite enters the host through mucosal surfaces or breaks in the skin, typically via contamination with feces of infected triatomine bugs. Once inside the host, the infective trypomastigote form invades a wide range of nucleated cells by exploiting various endocytic and membrane fusion pathways.^21^ After internalization, the parasite escapes the parasitophorous vacuole and transforms into the replicative amastigote form within the cytoplasm. *T. cruzi* has a remarkable ability to infect all nucleated cell types, including muscle cells, epithelial cells, neurons, and immune cells, contributing to the systemic nature and complexity of CD pathogenesis.^22^

Chronic Chagas disease (CCD) manifests in approximately 1/3rd of infected individuals and is the leading cause of infectious myocarditis in the world.^23^ This presents a uniquely challenging situation, where systemically immunosuppressive treatments for myocarditis can inhibit antiparasitic immune responses that prevent reactivation of dormant parasites in reservoir tissues.^24^ The low parasite burden typical of CCD combined with autoimmune mechanisms triggered by the infection, sustains immune activation and drives disease progression. Consequently, current treatments, benznidazole and nifurtimox, are more effective during the acute phase, lack immunomodulatory properties, and are associated with severe toxic effects.^25^ Therefore, new effective therapies are needed to both eliminate the parasite and modulate the inflammatory response to prevent irreversible tissue damage as well as to treat heart damage that is currently considered irreversible.^26,27^

In this context, we have successfully applied polymersomes (PS) to enhance intracellular drug delivery and reduce inflammation in both acute and chronic models of CD.^28,29^ Motivated by these results, we hypothesized that PS could improve the delivery of the **BTAc** molecule while modulating infection-induced inflammation. Thus, this study aimed to evaluate the antitrypanosomal activity of polymersomes loaded with the bithiophene compound, as well as to assess the host immune response and the trafficking of intact nanocarriers following cellular internalization. Together, these analyses provide insights into both the parasiticidal potential and the immunomodulatory effects of this nanoformulation, which are critical for controlling *T. cruzi* infection.

## Materials and Methods

### *In Silico* Properties

*In silico* predictions of **BTAc** were conducted using SwissADME free Web site.^30^ Molecular structures were drawn to generate SMILES codes, and the parameters such as physicochemical parameters, lipophilicity (LogP), water solubility, pharmacokinetics, and drug likeness were obtained.

### Synthesis of BTAc

The bithiophene derivative was synthesized previously.^17^ For **BTAc**, the drug was dissolved in DMSO, with the concentration never exceeding 1% (v/v) to avoid any effects on parasites and cells.

### Synthesis of PEG-*b*-PPS

Copolymer blocks of PEG_17_-*b*-PPS_30_ were synthesized as earlier published.^10,31^ Briefly, PEG_17_-methoxy (Mn 750) was modified with mesylate groups, then reacted with thioacetic acid to yield PEG-thioacetate. This intermediate was activated with a base to generate a thiolate anion, which initiated the ring-opening polymerization of propylene sulfide. The resulting polymer was end-capped with benzyl bromide, producing PEG_17_-*b*-PPS_30_. Purified copolymers were analyzed via ^1^H NMR and block polymers purity was verified by gel permeation chromatography (GPC).^11,32^

### Nanocarrier Formation

Loaded and unloaded PS were formed using the thin-film hydration method.^32^ First, 20 or 30 mg of PEG_17_-*b*-PPS_30_ was dissolved in 250 mL of tetrahydrofuran (THF – Sigma), with or without 1 or 2 mg of **BTAc**, to produce **BTAc**-loaded PS (**BTAc**-PS) and unloaded/blank PS (B-PS), respectively. The organic solvent was evaporated in a desiccator for 24 h, hydrated with 1 mL of 0.01 M PBS, and then shaken for 24 h at 1500 rpm. Hydration of the dried film initiates the self-assembly of vesicular nanostructures. The PS nanocarriers were extruded three times through 0.2 μm PTFE membranes (Whatman). Unloaded drug was removed by Zeba Desalting Column (7K MWCO, ThermoFisher Scientific), following the manufacturer’s instructions. After optimization, PS were fabricated with 20 mg of copolymer with 2 mg of **BTAc** (**BTAc**-PS) or without drug for unloaded (blank) polymersomes (B-PS).

### Nanocarrier Characterization

PS were characterized by dynamic light scattering (DLS) for diameter, polydispersion index (PDI), and zeta potential assessment. Small-angle X-ray scattering (SAXS) and cryo-transmission electron microscopy (CryoTEM) were performed to analyze the vesicular shape of PS. Nanoparticle tracking analysis (NTA) was performed using a NanoSight 300 (Malvern) to characterize the particle size distribution and quantify particle concentration. Detailed methods are described in the Supporting Information (Methods S1–S3). The encapsulation efficiency (EE%) and drug loading (DL%)^33^ were determined by high-performance liquid chromatography (HPLC). Briefly, for quantification, the samples were diluted in acetonitrile (ACN), agitated for 24 h, frozen at −80 °C and ultracentrifuged at 17000 g for 15 min. The HPLC methodology is depicted in the Supporting Information (Figure S1), and the equations are described at the Supporting Information (Experimental Section S4). Drug release was assessed over a 7-day period in aqueous solution, with 500 μL of **BTAc**-PS diluted in 4.5 mL of PBS 0.01 M or PBS+FBS 10% at 37 °C under magnetic stirring. The samples were collected at different time points (0.33, 0.66, 4, 6, 8, 24, 48, 72, and 168 h), purified using Zeba desalting columns, and the EE% was performed by HPLC as described above.

### Shelf Life Stability

PS stability was evaluated after 60 days of storage without stirring. Briefly, the DLS and EE% of **BTAc**-PS were analyzed at different time points (0, 15, 30, 45, and 60 days) under different storage temperatures (4, 25, or 37 °C). At each time point, aliquots were collected, and all procedures were performed exactly as described above.

### Cell Lines and Parasite Culture

*T. cruzi*: Y, CL Brener *luc+* (BEI Resources NR-49161) and Brazil *luc+* (BEI Resources NR-40347) strains were used. Trypomastigote forms were collected from the supernatants of LLCMK_2_ cell monolayers (kidney epithelial cells from *Macaca mulatta*, CCL-7; ATCC, Rockville, MD) previously infected with blood-derived trypomastigotes. LLCMK_2_ and RAW 264.7 macrophages were maintained in DMEM supplemented with 2 mM l-glutamine, 1% streptomycin and penicillin, 10% heat-inactivated FBS, and buffered with sodium bicarbonate in a 5% CO_2_ atmosphere at 37 °C.

### *In Vitro* PS Activity against *T. cruzi* and Cytotoxicity to Macrophages

To assess the antiprotozoal activity, intracellular amastigotes were obtained by infection of RAW 264.7 macrophages (5 × 10^5^ cells/mL) with trypomastigotes (MOI 10:1). Briefly, the infected macrophages were treated with different concentrations of **BTAc**-PS, B-PS, unloaded **BTAc** (free **BTAc**) and benznidazole (BZN) and incubated for 48 h, at 37 °C and 5% CO_2_. After incubation, the effect against CL Brener and Brazil strains were analyzed using the luciferase activity quantified by Pierce Firefly Luc One-Step Glow Assay Kit (ThermoFisher Scientific) to determine the trypanocidal activity as previously described.^28^ The antiamastigote assay against Y strain was analyzed by staining with Giemsa and counting the percentage of infected cells as previously described.^34^ The concentrations used ranged from 200 to 6.25 μg/mL and the inhibitory concentration for 50% of parasites (IC_50_) was calculated by nonlinear regression using GraphPad Prism 5. BZN (10–0.31 μg/mL) was used as a positive control.

The cytotoxicity of **BTAc** against RAW 264.7 macrophages was assessed using the MTT colorimetric assay^35^ as previously described.^36^ Briefly, macrophages (5 × 10^5^ cells/mL) were seeded in 96-well plates and incubated for 24 h to allow monolayer formation. Subsequently, various concentrations of **BTAc**-PS, B-PS, free **BTAc**, and BZN were added and incubated for 48 h at 37 °C with 5% CO_2_. After incubation, MTT solution (2 mg/mL) was added, and plates were incubated for an additional 4 h. The resulting formazan crystals were dissolved in 150 μL of DMSO, and absorbance was measured at 570 nm using a spectrophotometer. Concentrations ranged from 600 to 18.75 μg/mL, and the 50% cytotoxic concentration (CC_50_) was determined by nonlinear regression analysis using GraphPad Prism 5. BZN (200–6.25 μg/mL) was used as positive control.

The selectivity index (SI) was calculated as the ratio of CC_50_ to IC_50_ (SI = CC_50_/IC_50_).

### Nanocarrier Uptake by Macrophages and Payload Release Monitored Via FRET Confocal Microscopy

Förster resonance energy transfer (FRET) was used to measure the nanocarrier uptake and payload release by macrophages. PS were fabricated with (2Z)-2-[(E)-3-(3,3-dimethyl-1-octadecylindol-1-ium-2-yl)­prop-2-enylidene]-3,3dimethyl-1-octadecylindole (DiI) and 1,1′-dioctadecyl-3,3,3′,3′-tetramethylindodicarbocyanin (DiD) as a pair of dyes known by promoting FRET phenomena.^37^ DiI (0.012 mg/mL) and DiD (0.053 mg/mL) were loaded combined (DiI 1:4 DiD) or separately in 20 mg of copolymer to produce DiI-PS, DiD-PS and FRET-PS (dyes combined), as described above. DLS was used to assess particle diameter and PDI. The encapsulation efficiency was determined by fluorescence at λex:543/λem:570 nm for DiI and λex:630/λem:670 nm for DiD. FRET fluorescence was detected in λex:543/λem:670 nm. FRET was validated by fluorescence scanning of intact PS dissolved in aqueous solution or dissociated PS dissolved in an organic solvent THF (λex:543/λem:600–700 nm).

RAW 264.7 macrophages (1.25 × 10^5^ cell/mL) were seeded in 4-chamber coverglass bottom Petri dishes and incubated for 24 h at 37 °C and 5% CO_2_. Afterward, the macrophages were infected with CL Brener *luc+* strain (MOI 10:1) and incubated for additional 24 h at the same conditions, as published before.^28^ Then, the cells were treated with 20 μL of different PS (0.4 mg/mL of copolymer) and incubated for 2, 4, or 24 h. After the incubation period, noninternalized nanocarriers were removed by washing with PBS. Cells were then fixed with 2% paraformaldehyde and permeabilized with 0.1% Triton X-100 for 20 min. For immunolabeling, samples were incubated for 1 h at room temperature with goat antiluciferase polyclonal antibody (Promega) diluted 1:500 in 1% bovine serum albumin (BSA; Sigma). Subsequently, samples were incubated with Alexa Fluor 488-conjugated donkey antigoat IgG (1:1000; Invitrogen) diluted in 1% BSA for 1 h at room temperature. Nuclei were stained with NucBlue (Invitrogen), following the manufacturer’s instructions. Imaging was performed using a Leica TCS SP8 Confocal Microscope under the standard FRET protocol.

### Ultrastructural Assessment

To assess the alterations on intracellular amastigotes and host-cells, RAW 264.7 (5 × 10^5^ cell/mL) macrophages were seeded in 125 cm^3^ flasks or 24-well plates, for electron microscopy (EM). The monolayers were infected with trypomastigotes of Y strain (MOI 10:1) and incubated for 24 h at 37 °C and 5% CO_2_. After the infection period, the noninternalized parasites were washed out and the cells were treated with respective IC_50_ of **BTAc**-PS (30.68 μg/mL of drug/0.92 mg/mL of copolymer), free **BTAc** (40.13 μg/mL of drug), BZN (4.24 μg/mL of drug) and the equivalent of copolymer of IC_50_ for B-PS (0.92 mg/mL of copolymer) and incubated for 48 h.

For EM, the cells were fixed in 2.5% glutaraldehyde in 0.1 M sodium cacodylate buffer. For transmission electron microscopy (TEM), the samples were postfixed in a solution that contained 1% osmium tetroxide, 0.8% potassium ferrocyanide, and 5 mM calcium chloride. The parasites were dehydrated in an acetone series and embedded in Polybed 81 resin for 72 h at 60 °C. Ultrathin sections were obtained in an ultramicrotome Leica EM UC7 and stained with 5% uranyl acetate and lead citrate.^38^ The images were captured in a JEOL JEM 1400 transmission electron microscope. For scanning electron microscopy (SEM), the samples were dehydrated in a graded series of ethanol, critical-point dried in CO_2_, coated with gold and analyzed in a Shimadzu SS-550 scanning electron microscope.

### Biochemical Measurements

For the assessment of biochemical parameters, including cytokines, reactive oxygen species (ROS), and nitric oxide (NO), cells were seeded in 96-well black plates under the same conditions described above in the *Ultrastructural Assessment* section. For ROS, the cells were washed with PBS and loaded with 10 μM of a pro-fluorescent dye H_2_DCFDA in absence of light for 45 min.^36^ Reactive oxygen species were detected at λex: 488 nm and λem: 530 nm in a Victor X3 spectrofluorometer (PerkinElmer, Waltham, MA).

For intracellular NO, the cells were loaded with DAF-FM-DA at 1 μM and incubated for 30 min in the absence of light. Then, the cells were washed with PBS and the DAF-FM fluorescence was measured at λex:495 nm and λem: 515 nm.^36^

For cytokines assessment, 50 μL of the supernatant was collected, and the cytokines determination was made with BD CBA Mouse Inflammation Cytokine Kit (Becton Dickinson, Franklin Lakes, NJ) according to the manufacturer’s instructions.^39^ Data acquisition was performed on a flow cytometer FACSCalibur equipped with CellQuest software. The analysis was made using the open-source Floreada software.

### Statistics

The data are expressed as the mean and standard deviation from at least three independent experiments. Data were analyzed using one or two-way analysis of variance (ANOVA), followed by Tukey’s post hoc test to identify significant differences among group means. Values of *p* < 0.05 were considered statistically significant. The statistical analyses were conducted using GraphPad Prism 5 software (San Diego, CA).

## Results

### Molecules

The successful synthesis of the PEG_17_-*b*-PPS_30_ copolymer (Figure 1A) was confirmed by ^1^H NMR spectroscopy, while the diblock copolymer structure and purity were verified by GPC analysis, which indicated a uniform molecular weight distribution, as evidenced by the single, narrow peak shown in Figure S2. *In silico* ADME-Tox analysis indicated that **BTAc** is a moderately lipophilic compound (LogP = 3.67) with favorable drug-likeness, showing no violations of Lipinski’s or Veber’s rules and no Pan Assay Interference compounds (PAINS) alerts (Figure 1B and Figure S3A). Although **BTAc** is predicted to interact with certain cytochrome P450 enzymes (CYP1A2, CYP2C19, and CYP2C9) (Figure S3A), it demonstrated high predicted gastrointestinal absorption and no blood–brain barrier (BBB) permeation, suggesting good oral bioavailability with minimal risk of neurological side effects (Figure S3B). Furthermore, **BTAc** is not a predicted substrate of P-glycoprotein (P-gp), which may contribute to prolonged intracellular retention.

**Figure 1:**
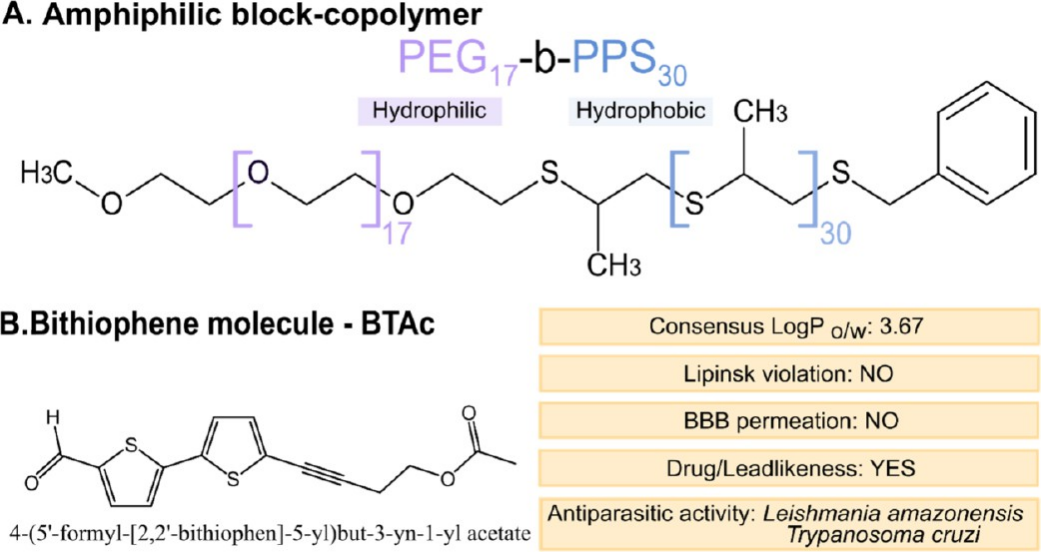
Schematic representation of the molecules used in this study. (A) Amphiphilic copolymer poly­(ethylene glycol)-*block*-poly­(propylene sulfide) (PEG-*b*-PPS) used for polymersome formation. (B) Bithiophene-based drug candidate encapsulated within polymersomes (PS), along with its key physicochemical properties.

### Nanocarrier Preparation

PS were prepared by thin-film hydration, as shown in Figure 2A. The ideal drug-to-copolymer ratio was chosen based on the particle diameter measurements, PDI, drug encapsulation and loading efficiency. Although the three drug:copolymer ratios showed significant differences in size, all formulations exhibited suitable dimensions for the intended purpose of this study: macrophage internalization. The PDI values of the 2:20 and 2:30 drug:copolymer ratios were significantly lower than that of the 1:20 ratio (Figure 2B), indicating a more homogeneous nanocarrier suspension. Although the 1:20 ratio showed a higher encapsulation percentage (Figure 2C), it resulted in a lower amount of drug encapsulated, as indicated by the drug loading (Figure 2D). The 2:20 ratio was considered the most suitable as it exhibited the highest drug loading while maintaining an appropriate particle size and homogeneity. Considering these results, the 2:20 ratio was selected for the preparation of **BTAc**-PS.

**Figure 2:**
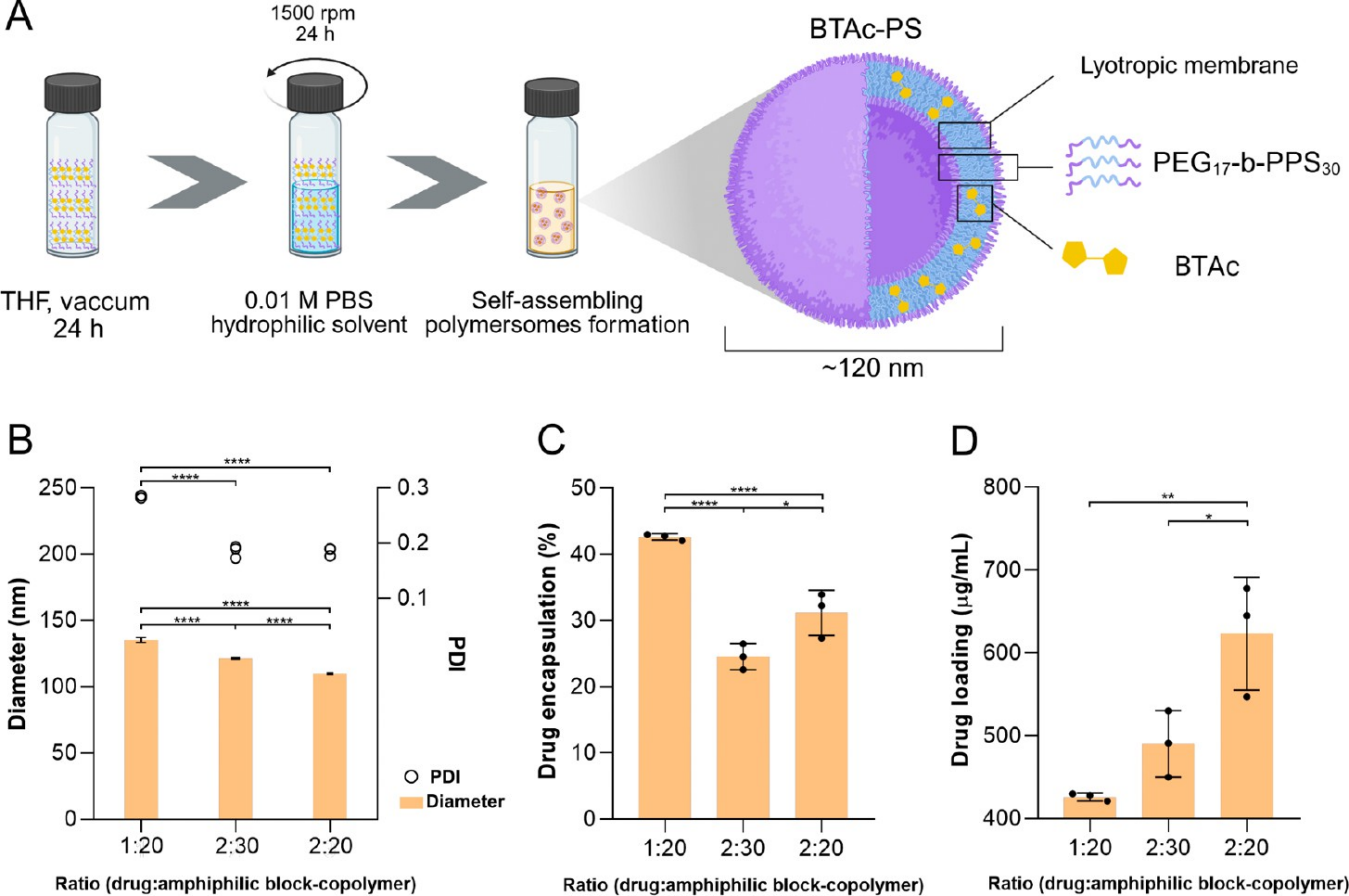
Nanocarrier formation and optimization. (A) Self-assembling schematic illustration of polymersomes encapsulating a bithiophene derivative BTAc (**BTAc**-PS). (B) Dynamic light scattering (DLS) of different ratios of drug and copolymer, showing size average (left axes) and polydispersion index (right axes). (C) Encapsulation efficiency comparison between different ratios of drug and copolymer. (D) Drug loading comparison between different ratios of drug and copolymer. One-way ANOVA with Tukey’s post-test was used for multiple comparisons (*n* = 3). *p < 0.05, **p < 0.005, ***p < 0.0005, ****p ≤ 0.0001.

### Physicochemical Characterization of Nanocarriers

After the optimization, all PS were produced using the 2:20 drug:copolymer ratio. No significant difference regarding the diameter and PDI were observed for **BTAc**-PS and B-PS. By HPLC analysis, encapsulated PS showed an average encapsulation efficiency of 33.82% for **BTAc**, resulting in a nanocarrier suspension containing an average drug concentration of 676.4 μg/mL (Figures 3A and S1).

**Figure 3:**
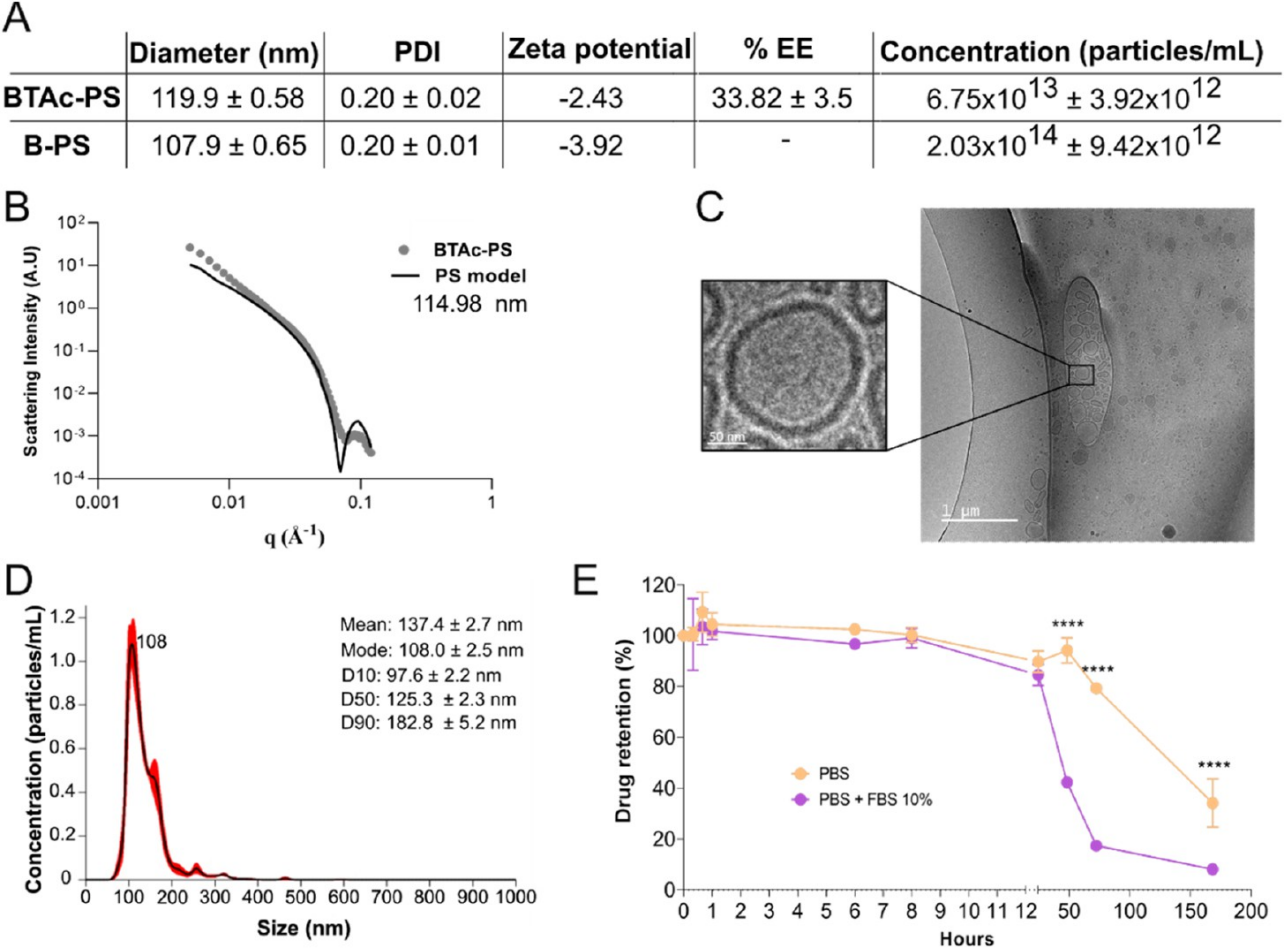
Physicochemical characterization of PS nanocarriers. (A) A summary of PS properties (PDI: polydispersity index; EE: encapsulation efficiency). (B) Small-angle X-ray scattering (SAXS) of **BTAc**-PS fitting in a vesicle model. (C) Representative cryo-TEM of **BTAc**-PS to confirm the vesicle morphology. (D) NanoSight nanoparticle analysis of size distribution and concentration of PS diluted in water. (E) Drug release profile of **BTAc**-PS under different conditions (PBS and PBS+FBS 10%) after 7 days at 37 °C. One-way ANOVA (*n* = 3) with Tukey’s post-test was used for multiple comparisons ****p ≤ 0.0001.

The vesicular structure of **BTAc**-PS was confirmed by SAXS and cryo-TEM analyses. The SAXS profile was consistent with a vesicular model, indicating a size of approximately ∼115 nm, while the bilayer membrane was visible in the cryo-TEM images (Figure 3B,C). NTA revealed a mean particle size of approximately 137 nm, with 10%, 50%, and 90% of particles below 96.7, 125.3, and 182.8 nm, respectively. NTA also revealed a particle concentration of 6.75 × 10^13^ particles/mL (Figure 3A–D).

Drug release studies were performed in PBS and PBS supplemented with FBS at 37 °C under agitation. No drug release was observed in either solution until 24 h of incubation at 37 °C under agitation. However, after this period, the FBS-containing PBS showed a significantly more rapid release, with 50% of the drug released at 48 h and 85% at 72 h. In contrast, the PBS solution demonstrated greater stability, with only 20% release at 72 h and 80% after 7 days of incubation at 37 °C and agitation (Figure 3E).

To evaluate the shelf life stability of **BTAc**-PS, three different storage temperatures were tested: 4 °C, 25 °C, and 37 °C with no magnetic stirring. The particle diameter and dispersity remained stable over 60 days at all temperatures with no significant difference (Figure 4A). However, PS containing **BTAc** stored at 4 °C released the drug significantly faster than at 25 and 37 °C. At 4 °C, 30% of the drug was released within the first 15 days, reaching a maximum release of 47%. In contrast, no significant release was observed from PS stored at 25 and 37 °C during this same period. By day 30, drug release reached 19% and 33% at 25 °C and 37 °C, respectively. After 60 days, all three storage conditions resulted in approximately 50% drug release (Figure 4B).

**Figure 4:**
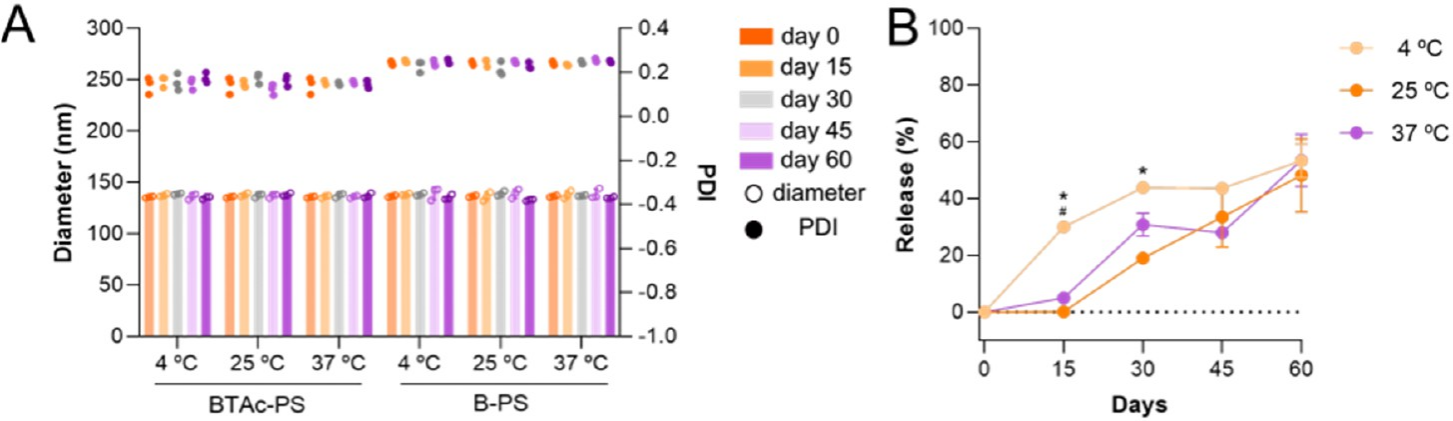
Stability of nanocarriers. (A) Diameter (left axes) and PDI (right axes) stability of **BTAc**-PS after 60 days. (B) Drug release stability of **BTAc**-PS after 60 days. One-way ANOVA (*n* = 3) with Tukey’s post-test was used for multiple comparisons with *p < 0.05 compared to 25 °C; ^#^ *p* < 0.05 compared to 37 °C.

### Biological Activity of BTAc-PS against *T. cruzi*

The antitrypanosomal activity of the loaded and unloaded nanocarriers, as well as free **BTAc** was evaluated against intracellular amastigotes of *T. cruzi* strains using RAW 264.7 macrophages as host cells. The IC_50_ was calculated based on the amount of encapsulated drug. **BTAc**-PS showed the most effective activity against CL Brener amastigotes, with the lowest IC_50_ value of 6.17 μg/mL. Brazil and Y strains were more resistant, with IC_50_ values of 24.01 and 30.68 μg/mL, respectively. B-PS exhibited significantly higher IC_50_ values than its respective loaded counterpart, indicating that the unloaded PS had no activity against intracellular amastigotes. In all cases, **BTAc**-PS exhibited lower IC_50_ values than the free drug (free **BTAc**) (Table 1).

**Table 1:** Activity of **BTAc**-Loaded Polymersomes, Benznidazole, and Free **BTAc** against Intracellular Amastigotes of *T. cruzi* and Cytotoxicity

IC_50_ (μg/mL)				
*Trypanosoma cruzi* strain	BTAc-PS	B-PS^a^	Free BTAc	BZN
CL Brener	6.17 ± 1.64**	1045.45 ± 198.48^a*^	66.68 ± 2.51	N/A
Brazil	24.01 ± 0.73^****#^	>3000^a****####^	116.33 ± 17.32^####^	0.68 ± 0.07****
Y	30.68 ± 12.7^**####^	>3000^a****####^	40.13 ± 2.29^###^	4.24 ± 0.15***

mammalian cells	CC_50_ (μg/mL)			
LLCKM_2_	480 ± 75.64^****####^	>15,000^a****####^	194.82 ± 7.35	194.86 ± 4.96
RAW 264.7	153.94 ± 3.8****	>20,000^a****####^	390.26 ± 22.63****	173.49 ± 2.77^####^
Red blood cells	>1000	>20,000^a^	>1000	>1000

a Values referring to copolymer concentration; IC_50_: inhibitory concentration for 50% of parasites; CC_50_: cytotoxic concentration for 50% of cells. **BTAc**-PS: polymersomes loaded with **BTAc**. B-PS: unloaded polymersomes (blank); BZN: benznidazole. Statistical significance was determined by two-way ANOVA (*n* = 3) followed by Tukey’s multiple comparisons test. Data are represented as mean ± SD *p < 0.05, **p < 0.005, ***p < 0.0005, ****p ≤ 0.0001 if compared to free drug and ^#^ *p* < 0.05, ^##^ *p* < 0.005, ^###^ *p* < 0.0005, ^####^ *p* ≤ 0.0001 if compared to benznidazole.

The cytotoxic concentration (CC_50_) of **BTAc**-PS on epithelial LLCMK_2_ cells was higher than that of the free drug, indicating lower toxicity. However, in RAW264.7 macrophages, the CC_50_ of **BTAc**-PS was lower than free **BTAc**. No hemolytic activity was observed for either the loaded or unloaded PS formulations, as well as for the free drug and BZN (Table 1). Based on the IC_50_ values, the selectivity index (SI) of **BTA**c-PS was highest for the CL Brener strain, followed by the Brazil and Y strains (Table 2). For CL Brener strain, the SI was significantly higher than that of the free drug. For the last two strains, no significant differences were observed when compared with the free drug.

**Table 2:** Selectivity Index of Polymersomes, and free **BTAc**, Considering the Ratio between CC_50_/IC_50_, and Toxicity to Mammalian Cells^a^

RAW264.7	SI		
*Trypanosoma cruzi* strain	BTAc-PS	Free BTAc	BZN
**CL Brener**	26.99 ± 7.91**	5.86 ± 0.16	-
**Brazil**	6.41 ± 0.24^####^	3.42 ± 0.51^####^	280.60 ± 21.51****
**Y**	5.12 ± 0.13	9.77 ± 1.02^###^	41.75 ± 6.13***

a SI: selectivity index (the ratio between CC_50_/IC_50_). Statistical significance was determined by two-way ANOVA (*n* = 3) followed by Tukey’s multiple comparisons test. Data are represented as mean ± SD *p < 0.05, **p < 0.005, ***p < 0.0005, **** *p* ≤ 0.0001 if compared to free drug and ^#^ *p* < 0.05, ^##^ *p* < 0.005, ^###^ *p* < 0.0005, ^####^ *p* ≤ 0.0001 if compared to benznidazol.

### Cellular Uptake and Payload Release of Nanocarriers in Macrophages

Intracellular nanocarrier tracking was performed using FRET nanocarriers. The selected dye pair was DiI (donor) and DiD (acceptor) (Figure 5A), as neither dye overlaps with the fluorescent probes used to stain intracellular parasites. The FRET phenomenon occurs when both fluorophores are close, allowing the donor’s emission energy (DiI) to excite the acceptor fluorophore (DiD).^40^ In this scenario, the emission of the acceptor can be detected, indicating that the dyes are within approximately 10 nm of each other, thus enabling energy transfer. Conversely, if the payload is released or the nanocarrier dissociates, the fluorophores separate beyond this distance, preventing energy transfer and resulting in the loss of acceptor emission. Since the size and PDI of the fluorescent nanocarriers were similar to those of **BTAc**-PS (Figure 5B), DiI-PS, DiD-PS and FRET-PS were used to investigate the intracellular behavior of the PEG-*b*-PPS nanocarriers *in vitro*. FRET was first confirmed by fluorescence scanning at the respective excitation and emission wavelengths (Figure 5C). To validate the integrity of the FRET-PS system, fluorescence was measured in both aqueous and organic solvents. A loss of FRET signal in THF indicated nanocarrier disassembly, confirming the responsiveness of the system (Figure 5D).

**Figure 5:**
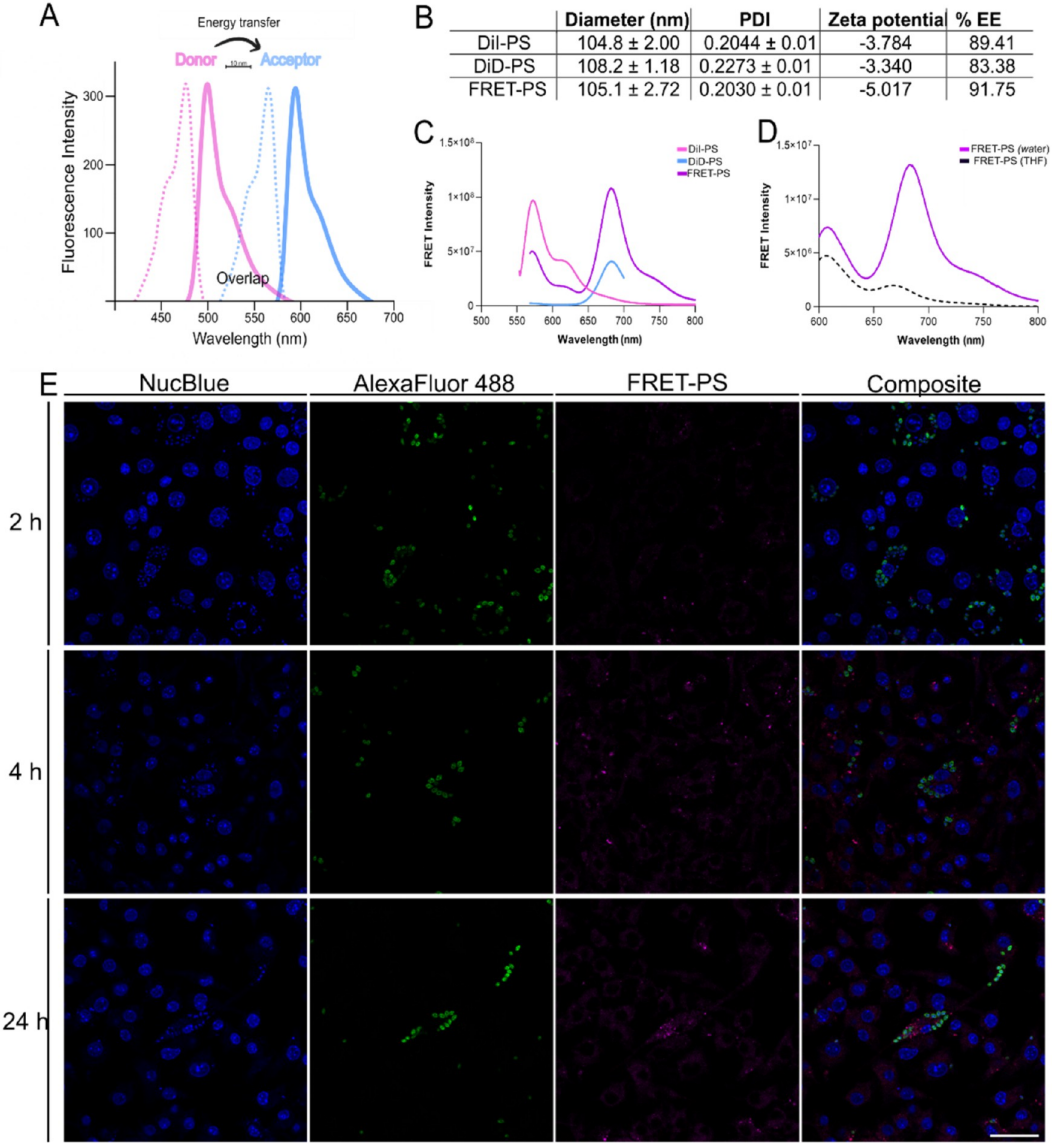
Intracellular delivery of polymersomes *in vitro* within macrophages RAW264.7 infected with *T. cruzi* CL Brener *luc+* strain. FRET polymersomes (FRET-PS) was produced with DiI as a donor dye and DiD as acceptor dye. (A) Schematic illustration of dye’s spectrum overlap and FRET occurrence. (B) Chart of physicochemical properties of fluorescent nanocarriers (PDI: polydispersity index; EE: encapsulation efficiency). (C) Spectral properties of DiI and DiD in the FRET system. DiI was detected at λex: 543 nm/λem: 570, DiD was detected at λex:630 nm/λem: 670 and FRET fluorescence was detected at λex: 543 nm/λem: 670 nm. (D) Validation of FRET system detected at λex:543 nm/λem: 670 nm. (E) Confocal images shows single channels NucBlue to indicate nucleus, antiluciferase antibody and Alexa 488 and antibody to indicate *T. cruzi* CL Brener *luc+*; nanocarriers detected by FRET fluorescence at λex: 543 nm/λem: 670 and merged channels. Scale bar: 30 μm.

Confocal microscopy analysis revealed that macrophages internalized the nanocarriers within 2 h of exposure, as indicated by the presence of small, concentrated fluorescent puncta, suggesting vesicular localization of the intact FRET-PS. After 4 h, the number of intracellular vesicles containing FRET-PS increased substantially. However, at 24 h postexposure, the concentrated vesicular signal was reduced, and the fluorescence appeared diffusely distributed throughout the cytoplasm, suggesting that intact PS reached the host cell cytosol (Figure 5E). Notably, no colocalization between the nanocarriers and intracellular amastigotes was observed (Supplementary video S1).

### Ultrastructural and Morphological Changes in Macrophages after Infection and Treatment with Nanocarriers

In the uninfected control group, macrophages exhibited typical healthy morphology, including numerous microvilli, a well-organized nucleus, and characteristic cell shape (Figure 6A1–A8). In contrast, cells treated with **BTAc**-PS or free **BTAc** showed a slight reduction in microvilli (Figure 6B3–D4). Interestingly, treatment with free **BTAc** frequently resulted in the appearance of binucleated cells and signs of chromatin condensation (Figure 6D1–D5). Chromatin condensation was also observed in macrophages treated with benznidazole (BZN). BZN treatment also resulted in a high number of cytoplasmic vacuoles and an abundance of vesicles surrounding the cells (Figures 6E1–E8).

**Figure 6:**
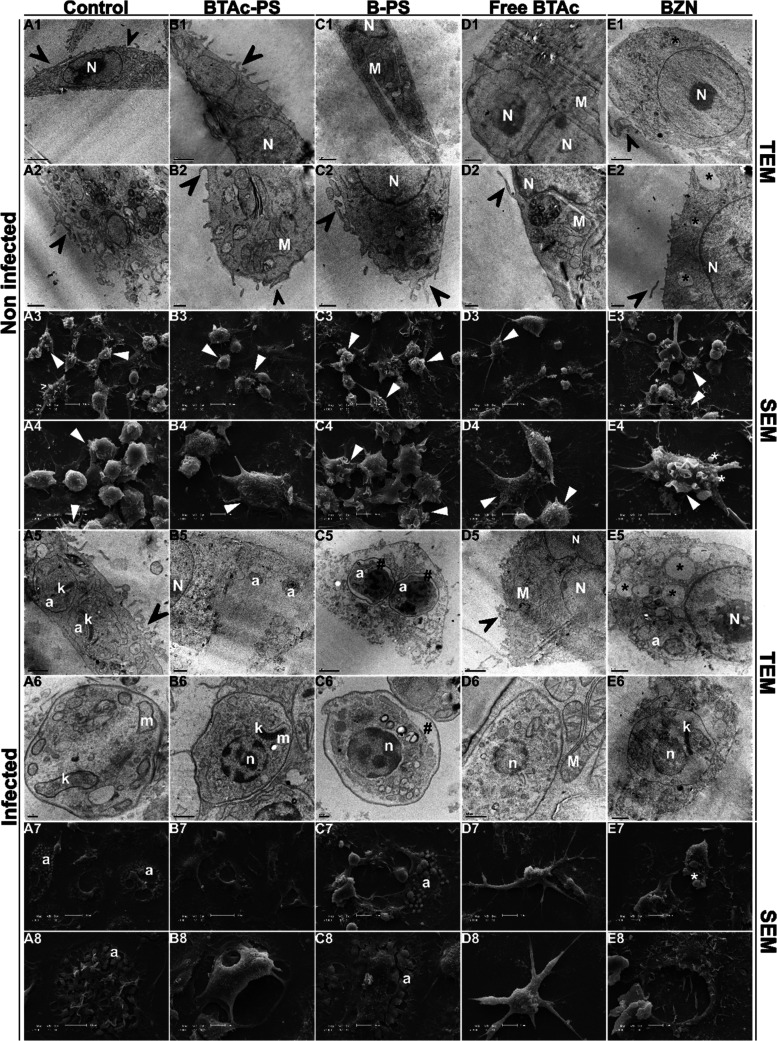
Transmission and scanning electron microscopy of RAW 264.7 macrophages infected or not with *Trypanosoma cruzi* and treated for 48 h. (1–4) Noninfected RAW 264.7 macrophages; (5–8) RAW 264.7 macrophages infected with *T. cruzi* Y strain. (A) untreated cells; (B) cells treated with **BTAc**-PS at 30.68 μg/mL of drug/0.92 mg/mL of copolymer; (C) cells treated with B-PS at 0.92 mg/mL of copolymer; (D) cells treated with **BTAc**-free at 40.13 μg/mL; (E) cells treated with benznidazole at 4.24 μg/mL. (N) macrophage’s nucleous; (n) parasite’s nucleus; (M) macrophage’s mitochondria; (m) parasite’s mitochondria; (a) amastigotes; (k) kinetoplast; (arrows) microvilli; (black*) vacuoles; (white*) vesicles; (#) membrane detachment; Scale bar: **20 μm** = A7, B7; **10 μm** = A3, A8, B3, C3, C7, D3, D7, E3, E7; **5 μm** = A4, B4, B8, C4, C8, D4, D8, E4, E8; **2 μm** = A1, B5, D1, D5, E1; **1 μm** = A2, A5, B1, C1, C2, C5, E2, E5; **0.5 μm** = B2, B6, D2, D6, E6; **0.2 μm** = A6, C6.

In infected macrophages, similar morphological alterations were observed across treatments; however, the number of intracellular amastigotes varied. Cells treated with **BTAc**-PS, free **BTAc**, or BZN showed a marked reduction in parasite load, with amastigotes rarely observed (Figure 6B7–E8). In contrast, control and B-PS-treated macrophages displayed high levels of infection (Figure 6A7–C8). Notably, amastigotes within macrophages treated with **BTAc**-PS, free **BTAc**, or BZN exhibited disrupted chromatin organization (Figure 6B5–E6), and those treated with **BTAc**-PS also presented mitochondrial swelling (Figure 6B5–B6). Additionally, in both macrophages and amastigotes exposed to B-PS, detachment between the cytoplasm and plasma membrane was observed (Figure 6C1–C6).

### Cytokines and Biochemical Parameters

Surprisingly, the free **BTAc** reduced significantly the levels of pro-inflammatory cytokines in both infected and noninfected macrophages. In the infected group, tumor necrosis factor-α (TNF-α) levels were reduced significantly by 41.92% following free **BTAc** treatment compared to the control group. When compared to the control and noninfected group, the reduction was 38.27%. Furthermore, a significant reduction in TNF-α was observed between the infected and noninfected groups treated with the free **BTAc**, with a 29.50% decrease in concentration of cytokines in infected cells (Figure 7A).

**Figure 7:**
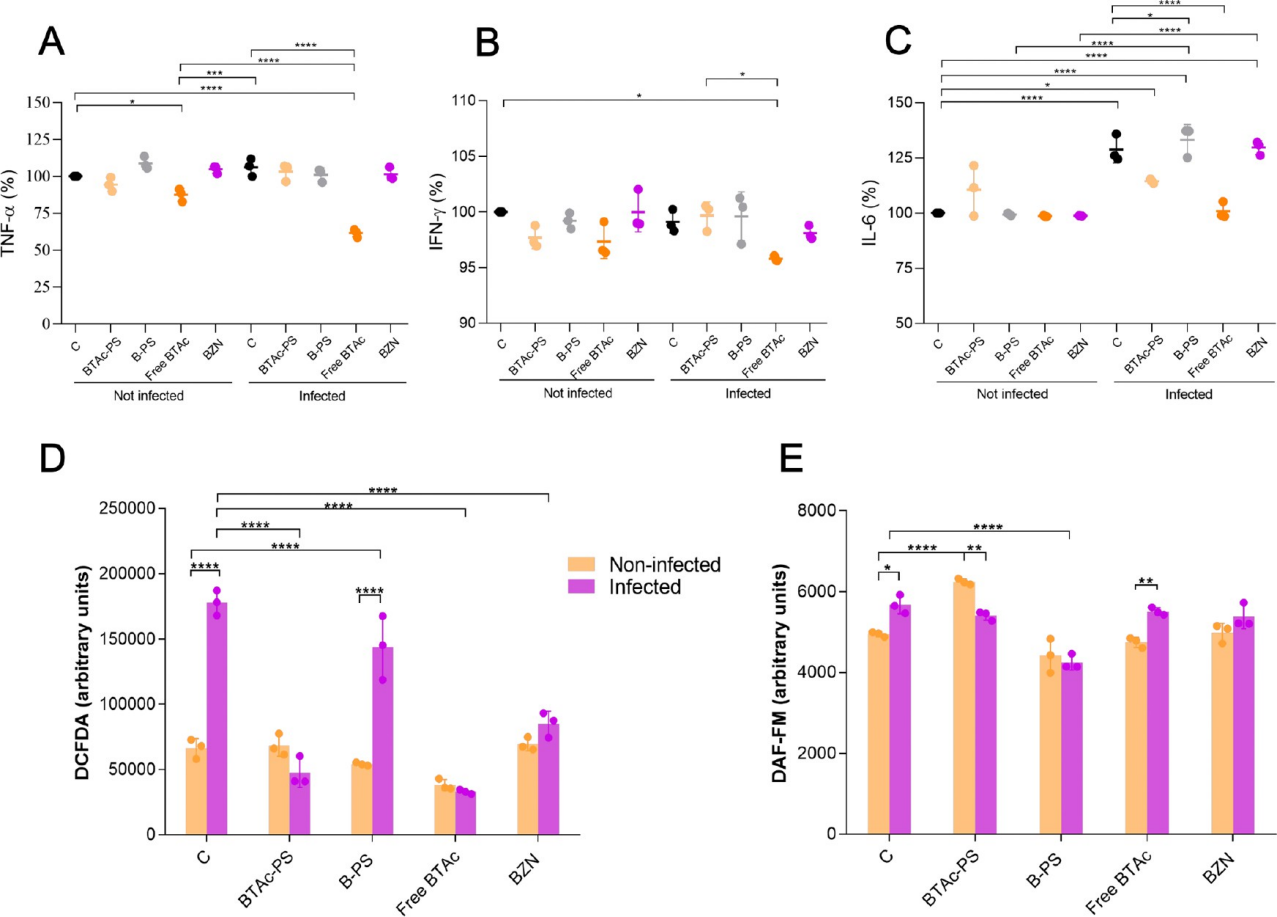
Biochemical response of macrophages RAW264.7 infected or not with *Trypanosoma cruzi* Y strain following 48 h treatment with **BTAc**-PS (30.68 μg/mL of drug/0.92 mg/mL of copolymer), B-PS (0.92 mg/mL of copolymer), **BTAc**-free (40.13 μg/mL), and BZN (4.24 μg/mL). *In vitro* quantification of (A) tumor necrosis factor-α (TNF-α); (B) Interferon-γ (IFN-γ); (C) Interleukin-6 (IL-6) assessed by BD CBA mouse Th1/Th2/Th17 CBA Cytokine Kit; Statistical significance was determined by one-way ANOVA (*n* = 3) followed by Tukey’s multiple comparisons test. (D) Reactive oxygen species (ROS) assessed by H_2_DCFDA fluorescent dye. (E) Intracellular nitric oxide (NO) assessed by DAF-FM-DA fluorescence. Statistical significance was determined by two-way ANOVA (*n* = 3) followed by Tukey’s multiple comparisons test. Data are represented as mean ± SD *p < 0.05, **p < 0.005, ***p < 0.0005, **** *p* ≤ 0.0001.

Interferon-γ (IFN-γ) levels also decreased following treatment with the free drug **BTAc**. In the infected group, free **BTAc** treatment led to a significant reduction of 4.42% in IFN-γ levels compared to the control and noninfected group. Within the infected group, free **BTAc** resulted in a significant reduction of 3.83% of IFN-γ concentration compared to **BTAc**-PS treatment (Figure 7B).

Interleukin-6 (IL-6) showed increased levels after infection. Within the control groups, IL-6 levels rose significantly by 28.82% after infection. However, treatment with free **BTAc** reduced significatively IL-6 levels to baseline with a reduction of 21.62% compared to the infected control group, showing no significant difference compared to the control, noninfected group. Similarly, **BTAc**-PS treatment also reduced significantly IL-6 levels after infection, with a reduction of 11.11% compared to the infected control group. In contrast, neither the B-PS nor BZN were able to restore IL-6 levels postinfection (Figure 7C). IL-10, IL-17, IL-4 and IL-2 presented no significant differences (Figure S4).

ROS levels increased by 168.0% after infection in the control group. Treatment restored ROS levels to baseline, with significant reductions of 73.24, 81.42, and 52.10% following **BTAc**-PS, free **BTAc**, and BZN administration, respectively in the infected group. In contrast, B-PS treatment failed to restore ROS levels, which remained 117.03% higher than those of the noninfected, control group (Figure 7D).

Interestingly, NO levels increased significantly by 14.76% after infection in the control group. Treatment with **BTAc**-PS further elevated NO levels in both noninfected and infected groups, with significant increases of 26.17 and 9.32%, respectively, compared to noninfected control group. However, within the **BTAc**-PS-treated cells, the infected group exhibited significative lower NO levels, with 16.85% less than the noninfected group. In contrast, treatment with free **BTAc** resulted in a significant increase of 16.08% in NO levels following infection. B-PS do not show any difference compared to the noninfected control group but decreased significantly the NO levels by 10.69% after infection compared to the infected control group (Figure 7E).

## Discussion

The development of new strategies to treat patients with CD, particularly the chronic phase, remains largely neglected and demands greater investment and scientific attention. In this study, we successfully developed polymersomes loaded with the novel bithiophene-derived compound **BTAc**, which demonstrated *in vitro* antitrypanosomal activity. **BTAc** is a synthetic bithiophene molecule that has been proven to exhibit anti-*T. cruzi* activity, inducing parasite cell death through multiple pathways in different life cycle stages of parasite. However, **BTAc** showed the highest IC_50_ against amastigotes^17^ and given that *T. cruzi* intracellular amastigotes are the only developmental form capable of multiplying inside mammalian hosts, targeting these cells enhances the likelihood of effective intracellular drug delivery, disrupting the parasite’s replicative cycle.

*In silico* predictions also supported the safety and drug-likeness of this compound as a promising candidate for further development, especially when formulated in nanocarriers to overcome its hydrophobic character. The compound exhibited no neurotoxicity, no PAINS and complied fully with Lipinski’s rule of five and Veber’s criteria, suggesting good oral bioavailability^41^ when unloaded. The fact that this compound is not a glycoprotein substrate may contribute to its enhanced efficacy against intracellular forms of the parasite. Therefore, the PEG-*b*-PPS nanocarrier system holds strong translational potential for treating *T. cruzi* infections by combining intracellular delivery, passive targeting of myeloid cells, and immunomodulatory effects.^7^ Of note, PEG-*b*-PPS nanocarries can be surface engineered with targeting ligands for enhanced cell specificity and uptake.^42,43^

The nanocarriers of PEG-*b*-PPS containing **BTAc** exhibited a size range compatible with efficient uptake by immune cells, particularly macrophages and dendritic cells, which preferentially internalize particles around 100 nm. Additionally, their size favors uptake by lymph node–resident myeloid cells, which are known to internalize particles smaller than 200 nm in diameter.^44–46^ The polymeric system not only exhibited an ideal nanocarrier size but also demonstrated a low PDI, indicating a suitably monodisperse solution. This characteristic is a key advantage for drug delivery, as uniform nanocarriers are more efficiently recognized and internalized by antigen-presenting cells (APCs), whereas larger or highly polydisperse particles may escape immune surveillance and reduce therapeutic efficacy.^47^

After cellular uptake, PEG-*b*-PPS PS are susceptible to oxidation, which facilitates payload release. It has been demonstrated that a small degree of PPS oxidation to sulfoxide in PEG-*b*-PPS vesicles initiates the structural transition of PS into micelles, thereby triggering the release of encapsulated compounds. Furthermore, the oxidized aggregates exhibit hydrodynamic radii small enough to allow for renal clearance, contributing to the biocompatibility and safety of the system.^11,32^ Although it is known that PEG-*b*-PPS PS are internalized within 24 h and can migrate to the cytosol following oxidation of the PPS block, it remained unclear whether these nanoparticles could retain their structural integrity after escaping the endosomal compartments.^4,32^ In this context, our FRET results confirmed the hypothesis that PS remain structurally intact upon cellular uptake and release their payload exclusively within the intracellular environment. This confocal microscopy-based technique is well-established^48^ and has undergone several validation steps.^49^ The persistence of energy transfer signals after 24 h within the macrophage cytosol indicates that the fluorophores remained in proximity, a prerequisite for effective FRET, thus confirming the structural integrity of the nanocarriers in the cytoplasmic environment. Our research group previously quantified that 60% of the initial FRET signal was retained inside the cells after 24 h,^28^ supporting the conclusion that a fraction of the polymersomes successfully escaped the endosomal compartment intact and reached the cytosol, and released their payload. Here, by using confocal microscopy to detect a distinct FRET pair, we confirmed qualitatively that nanocarriers remained intact in the cytosol. This endosomal escape capability is a critical feature for achieving efficient cytosolic drug delivery.

Cell-free assays demonstrated that drug release occurred after 48 h under serum-like conditions simulated with FBS, likely due to protein corona formation, which may accelerate vesicle destabilization compared to PBS alone.^50^ In contrast, PEG-*b*-PPS nanocarriers diluted in PBS without protein interference remained more stable, supporting previous findings that drug release primarily occurs in intracellular environments via polymer oxidation, as its blood residence time is less than 24 h.^5,28^

*T. cruzi* is a genetically diverse protozoan parasite, with considerable strain-dependent differences in virulence and drug susceptibility.^51,52^ In this study, we evaluated the efficacy of our nanocarrier-based drug delivery system against three distinct *T. cruzi* strains: CL Brener, Y, and Brazil. Although only three strains from two different DTUs were tested in this study, the bithiophene compound showed broad effectiveness against *T. cruzi*, with its antiparasitic activity appearing to correlate with the BZN susceptibility of each strain. The SI for **BTAc**-PS revealed a favorable profile as it was at least five times more toxic to the parasites than to macrophages. Notably, CL Brener, a strain known to be susceptible to BZN, also showed high susceptibility to our nanocarrier system.^53^ In contrast, Brazil and Y strains, both partially resistant to BZN,^54^ demonstrated reduced sensitivity to the treatment, suggesting that resistance mechanisms may affect the **BTAc**’s efficacy. Although the SI of BZN is higher than that of the nanoparticle or the free drug, BZN is well-known to have limited efficacy in treating the CCD in humans and to lack anti-inflammatory effects in chronic patients.^55^

In the field of drug development, it is commonly assumed that an ideal drug should be effective against all species and/or strains. However, in *T. cruzi*, genetic variability occurs not only interstrains but also intrastrains, considering the distinct life-cycle forms of the parasite. In this context, and to standardize the experimental conditions and reduce variability, the Y strain was selected for macrophage infection in subsequent assays, as it is partially resistant to BZN and more virulent than the CL Brener strain,^53,56^ more prevalent in the Americas,^57^ and more frequently used for drug screenings.^58^

Electron micrographs were used to assess a broad range of cellular alterations, including ultrastructural and morphological changes. The presence of double nuclei in macrophages may indicate that **BTAc** could interfere in the cell cycle of macrophages. This interference could also be caused by infection, as previously documented^59^ or caused by the inflammation itself, as macrophages were observed polyploid.^60^ It is noteworthy that at IC_50_ concentrations, **BTAc** did not show cytotoxic for macrophages but resulted in intracellular amastigotes rarely observed in treated cells. However, structural alterations previously associated with **BTAc** exposure were identified in amastigotes at both treatments, free **BTAc** and **BTAc**-loaded nanocarriers.^17^

During acute *T. cruzi* infection, macrophages and dendritic cells (DCs) serve as the first line of defense by phagocytosing the parasites and initiating proinflammatory responses mediated by reactive oxygen species (ROS) and reactive nitrogen species (RNS), such as nitric oxide (NO) and peroxynitrite (ONOO^–^).^61^ These cytotoxic molecules, primarily induced by IFN-γ and TNF-α, are essential for controlling the parasite during the acute phase of infection. During the chronic phase, although parasites reside predominantly in tissues such as the heart and intestine, immune cells play an important role in regulating Th1 and Th17 responses, controlling parasite burden, and balancing inflammatory processes.^62,63^ For this reason, macrophages were chosen as the cellular model for infection in this study. Macrophage phenotypes are complex, plastic, and interchangeable in response to diverse environmental conditions but classically, M1 phenotype plays an inflammatory role, stimulating the expression of molecules such as iNOS, NADPH, NO, and ROS, and increasing the expression of MHC class II, CD86, AP-1, and NF-κB. They also produce cytokines such as TNF-α, IL-6, IL-12, and IL-1β. This response can lead to parasite destruction; however, in approximately 30–40% of patients, this balance is lost, leading to persistent inflammation and tissue damage characteristic of CCD.^61,64^

At the chronic phase, an imbalance between pro- and anti-inflammatory cytokines mechanisms may be involved in the autoimmune responses during the infection. A pro-inflammatory response is crucial for parasitic control and cytokines, such as IFN-γ, TNF-α and IL-6, are responsible for fighting against the infection. Chronic dysregulation and sustained overexpression of pro-inflammatory cytokines can drive tissue damage, promoting fibrosis, heart failure, and the establishment of immunopathogenic processes decades after the initial *T. cruzi* infection. IFN-γ is related to Th1 activation at the onset of infection but high levels of this cytokine during the chronic phase can affect the myocardium in some individuals. TNF-α is known to induce ROS formation and can lead to cardiomyocyte necrosis and apoptosis. IL-6 correlated with the development of thrombotic processes by increasing endothelial platelet adhesion and stimulating platelet production.^65,66^ Although this study employed an acute model, the results support further investigation in chronic models, as the immunomodulatory profile of **BTAc** may fulfill the need for dual antitrypanosomal and inflammatory modulation. Notably, **BTAc** was able to restore proinflammatory cytokine levels to baseline *in vitro*. This effect aligns with findings reported for other molecules bearing the thiophene scaffold, which have also demonstrated anti-inflammatory properties.^67,68^

Currently, BZN remains the standard treatment for Chagas disease, but BZN’s limited efficacy during chronic infection is a major drawback, given the severe side effects and its potential to exacerbate chronic inflammation.^69^ To address this limitation, we successfully developed a thiophene-based nanocarrier formulation with dual properties: antiparasitic and immunomodulatory. Importantly, our *in vitro* findings demonstrate that this formulation selectively reduces proinflammatory cytokines without affecting anti-inflammatory ones, thereby restoring immune balance in infected macrophages independently of ROS. However, the nanocarriers developed in this study were not only capable of modulating macrophage-mediated inflammation but, as evidenced by the IC_50_ values, **BTAc**-PS also enabled intracellular delivery of the candidate drug to the parasite’s amastigote form, which persists and multiplies within the mammalian host.

In light of the limited resources allocated to research on neglected tropical diseases such as Chagas disease, *in silico* approaches have been increasingly employed in drug discovery to optimize screening processes and reduce the time and costs associated with this field. Following the same rationale, *in vitro* models are an essential strategy to reduce early stage failure rates and optimize resource allocation. Therefore, it is worth noting that these results were obtained using *in vitro* macrophage cultures.

## Conclusion

Collectively, our data supports the potential of thiophene-loaded nanocarriers as a promising platform for controlling CD by simultaneously enhancing trypanocidal activity and controlling inflammation. Future studies should investigate the efficacy of **BTAc**-PS and free **BTAc** in animal models of CD, given the biological complexity of living organisms. Furthermore, combining immunomodulatory nanocarriers with BZN may offer a synergistic approach to simultaneously reduce parasite burden and mitigate immune-mediated tissue damage in chronic CD.

## Supplementary Material





## Notes

Z-stack confocal FRET video (AVI)
Small angle scattering methodology; cryogenic transmission electron microscopy methodology; nanoparticle tracking methodology; encapsulation efficiency and drug loading equations; high-precision liquid chromatography parameters; nuclear magnetic resonance and gas permeation chromatography of PEG-*b*-PPS; physicochemical and pharmacokinetics parameters obtained *in silico* of BTAc; cytokines profiles of infected and noninfected macrophages (IL-10, IL-17, IL-2 and IL-4). (PDF)
