# Supplementary material for: Cytosolic Delivery of a Bithiophene Derivative via Polymersomes Kills Trypanosoma cruzi Amastigotes and Modulates the Inflammatory Response
Source: ACS Appl Nano Mater. 2026 Feb 14;9(8):3689–703. doi: 10.1021/acsanm.5c05104 (PMC12954673; doi:10.1021/acsanm.5c05104)
Supplement: Supplementary file 2 [file an5c05104_si_002.pdf]

Supporting information

Cytosolic Delivery of a Bithiophene Derivative via Polymersomes Kills *Trypanosoma cruzi* Amastigotes and Modulates the Inflammatory Response

Rayanne Regina Beltrame Machado[1,3], Débora B. Scariot[3,4], Amanda Beatriz Kawano Bakoshi[1,3], El Hadji Arona Mbaye[3], Sultan Almunif[3], Swagat Sharma[3], Deysiane Lima Salvador[2], Caroline Fortuna[2], Sueli de Oliveira Silva Lautenschlager[1], Tânia Ueda Nakamura[1], Maria Helena Sarragiotto[2], Danielle Lazarin Bidóia[1], Evan Scott[3,4], Celso Vataru Nakamura[1*]

[1] Laboratório de Inovação Tecnológica no Desenvolvimento de Fármacos e Cosméticos, Departamento de Ciências Básica da Saúde, Universidade Estadual de Maringá (UEM), Maringá, Paraná, Brasil 87020-900
[2] Programa de Pós-graduação em Química, Departamento de Química, Universidade Estadual de Maringá (UEM), Maringá, Paraná, Brasil 87020-900
[3] Department of Biomedical Engineering, Chemistry of Life Processes Institute, Northwestern University, Evanston, IL, 60208, USA
[4] Department of Biomedical Engineering, NanoSTAR Institute, University of Virginia School of Medicine, Charlottesville, VA, 22903, USA

*Corresponding author at:
Maringá State University, Av. Colombo, n. 5790, Zona 7, CEP: 87020-900, Maringá, Paraná, Brazil.
Email: cvnakamura@gmail.com

Experimental Section

S1. SAXS: Smal angle X-ray scattering analysis was performed at beamline 16-ID at the Life Science X-Ray Scattering (LIX) at the National Synchrotron Light Source II (NSLS-II) of Brookhaven National Laboratory. The samples (40 µL, 5 mg/mL) were loaded into a custom-designed poly-methyl ethacrylate strip with a 2 mm-thick wells sealed between 100 µm Ultra-thin Glass (G-Leaf TM Nippon) (https://github.com/stupplab/SAXScells). Data acquisition was performed using a 15.5 keV collimated X-ray source, which generated a continuous q-range of 0.005-3.2 $Å^{-1}$. The momentum transfer vector q is defined as, where 2θ is the scattering angle. Background subtraction was performed using Lixtools (https://github.com/NSLS-II-LIX/lixtools). All data modeling was performed using SASview software.

S2. CryoTEM: For cryogenic transmission electron microscopy analysis, a 200-mesh lacey carbon grids were subjected to glow discharge for 30 s using a Pelco easiGlow glow-discharger, at 15 mA and a chamber pressure of 0.24 mbar. Then, a 5 µL aliquot of the nanoparticle was placed onto the grid, blotted for 5 s, and then subjected to plunge-freezing in liquid ethane using an FEI Vitrobot Mark IV. Afterward, the grids were inserted into a Gatan 626.5 cryo transfer holder and imaged at a temperature of −180 °C using a JEOL 1400 Flash TEM LaB6 emission TEM at 120 kV. Imaging data was collected with the Gatan Digital Micrograph software using a Gatan OneView 4k camera.

S3. Nanosight: For nanoparticle tracking analysis (NTA), polymersomes were diluted at 1:100000 , and analyzed in a Nanosight300 (Malvern) equipped with asCMOS camera and 488 nm blue laser using Nanosight NTA sotware version 3.4. Hardware and analysis settings were as follows: Laser type: Blue488, Camera Level: 13, Camera gain: 3, Slider Shutter: 1232, Slider Gain: 175, Frame Rate: 25.0 FPS, Temperature: 25.0°C, Detect Threshold: 3, Blur size: auto, Max Jump Distance: auto. Syringe speed: 20. 5 movies of 60 seconds were captured and the analysis were made in triplicate.

S4. EE and DL (%): The percentages of encapsulation efficiency (EE%) and drug loading (DL%) were calculated according to:

$$EE\ (\%) = \left(\frac{drug\ encapsulated}{total\ drug}\right) \times 100$$

$$DL\ (\%) = \left(\frac{drug\ encapsulated}{total\ polymer}\right) \times 100$$

Results

A

Column: Zorbax SB-C18 5µm (4.6 x 250 mm) – Agilent		
Mobile phase A : water		Oven: 40 ºC
Mobile phase B: acetonitrile		Wavelength: 320 nm
**Time**	**Flow (mL/min)**	**%B**
0.000	0.500	15.0
1.000	0.500	15.0
5.000	0.500	100.0
8.000	0.750	100.0
10.000	0.750	90.0
15.000	Stop run	

B

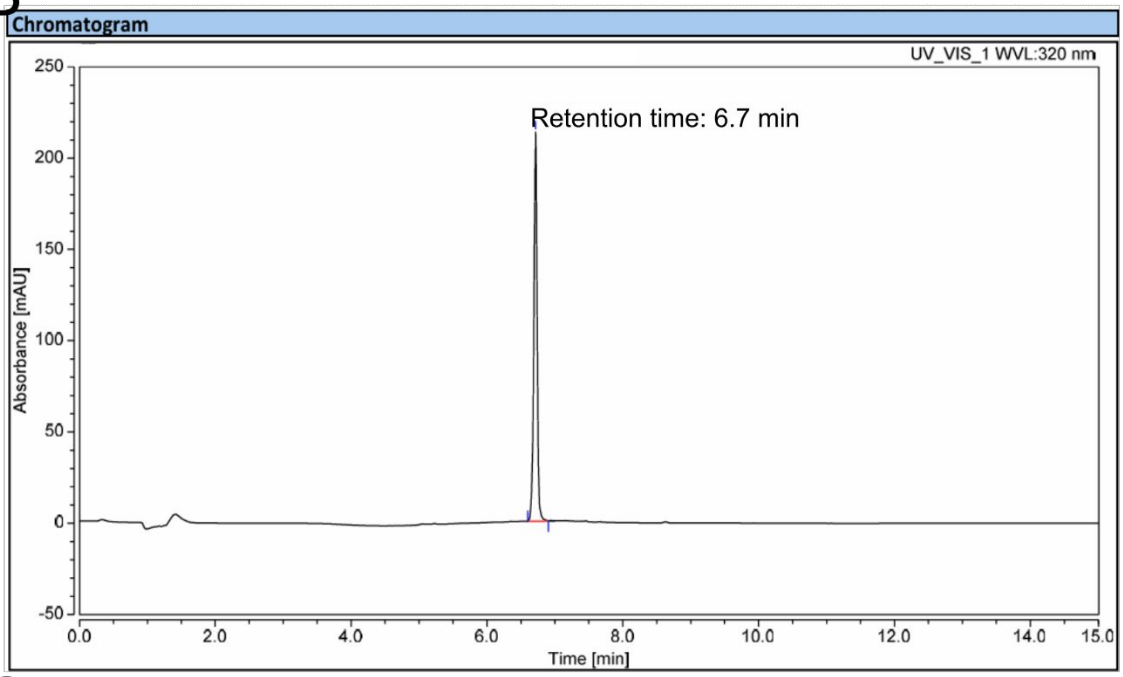

C

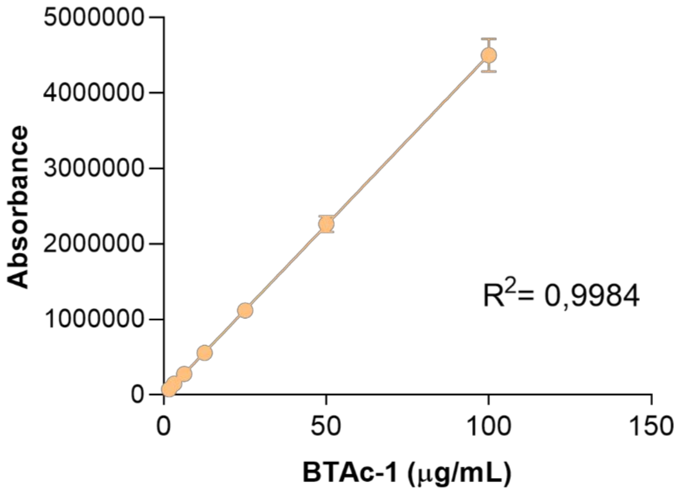

Figure S1: High precision liquid chromatography (HPLC) characterization. (A) Parameters used for **BTAc** detection. (B) **BTAc** chromatogram with retention time of 6.7 min, (C) Calibration curve of **BTAc** (n=3).

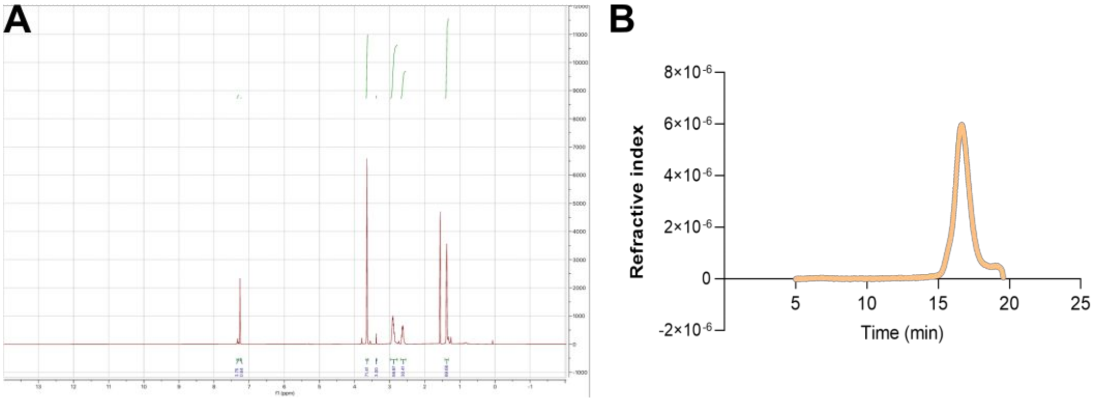

Figure S2: Characterization of co-polymer PEG-*b*-PPS. (A) ^{1}H Nuclear magnetic resonance of PEG-*b*-PPS. (B) Gas permeation chromatography (GPC) of PEG-*b*-PPS which revealed a single peak with a tetrahydrofuran mobile phase.

A

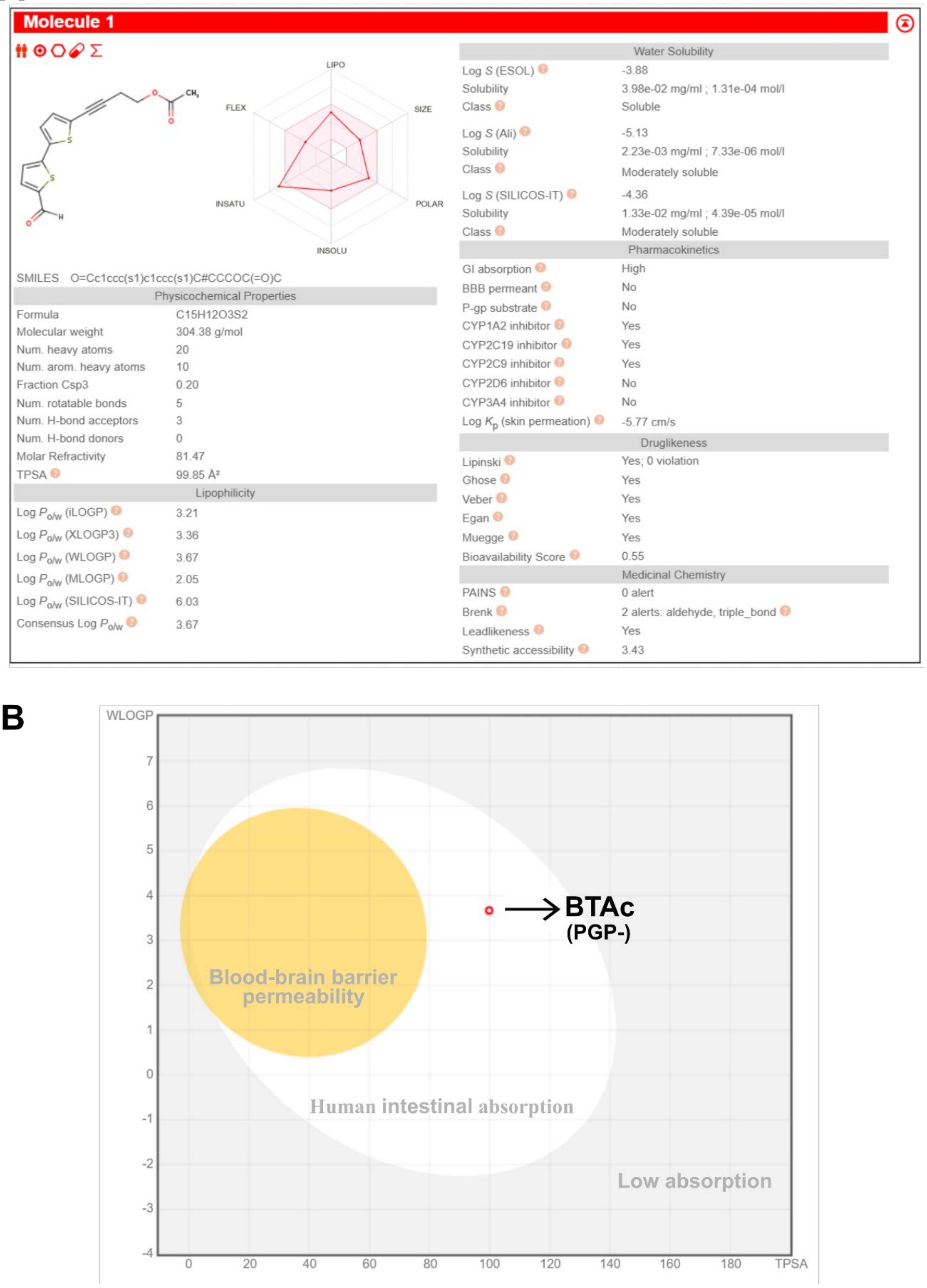

Figure S3: Physicochemical and pharmacokinetics properties of **BTAc**. (A) SwissADME chart containing all the predicted properties. (B) Boiled egg graph of BTAc absorption

parameters. PGP: P-glycoprotein; WLOGP: lipophilicity parameter; TPSA: topological polar surface area.

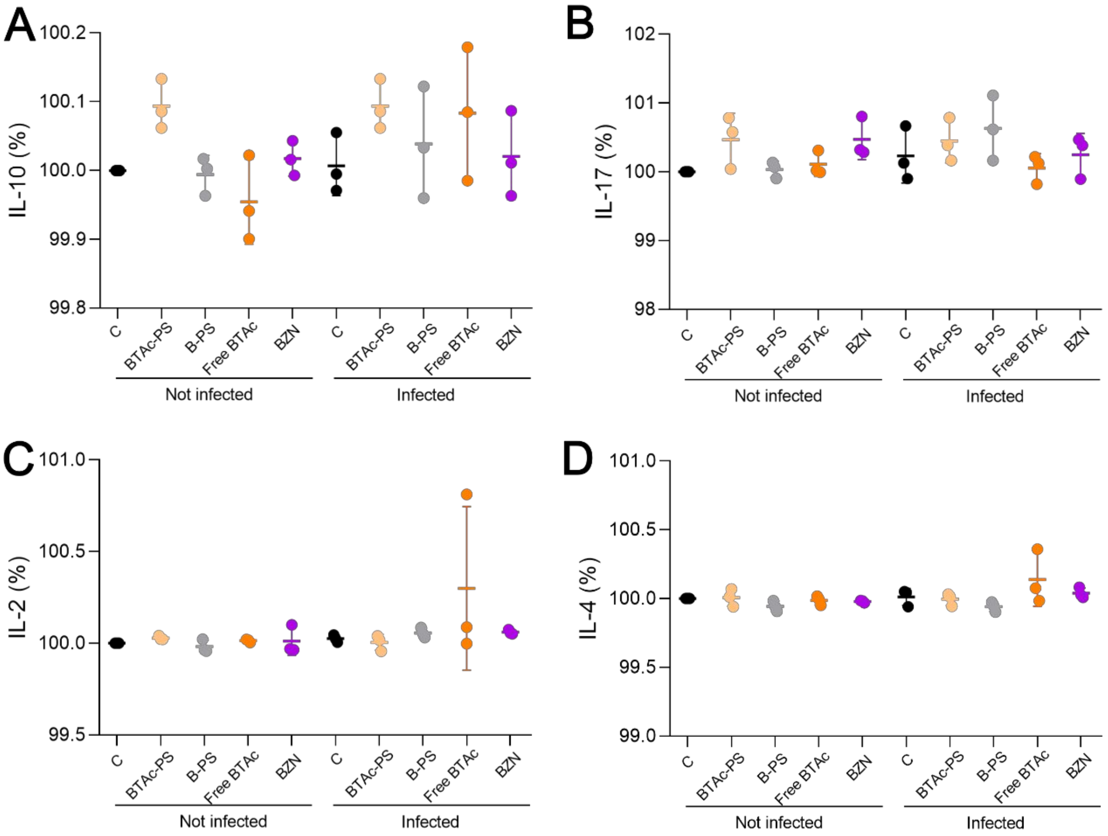

Figure S4: Cytokines production of macrophages RAW264.7 infected or not with *Trypanosoma cruzi* Y strain after treatment for 48 h with **BTAc**-PS, B-PS, free **BTAc** and benznidazole (BZN) with 30.68, 30.68, 40.13, and 4.24 µg/mL, respectively. (A) Interleukin-10 (IL-10) *in vitro* quantified; (B) Interleukin-17 (IL-17) *in vitro* quantified; (C) Interleukin-2 (IL-2) *in vitro* quantified; (D) Interleukin-4 (IL-4) *in vitro* quantified by CBA BD mouse Kit.

Video S1: The video is available at the following link: https://drive.google.com/drive/folders/1McEcI87cPJvb7XFaJ_vhbT38_hLA4dJJ?usp=drive_link
